# Investigating the impairment-performance relationship during competition in elite blind and partially sighted football

**DOI:** 10.3389/fspor.2025.1697819

**Published:** 2025-12-01

**Authors:** Harrison K. Leivers, Peter M. Allen, Matthew A. Timmis, Oliver R. Runswick

**Affiliations:** 1 Cambridge Centre for Sport and Exercise Sciences (CCSES), Anglia Ruskin University, Cambridge, United Kingdom; 2 Vision and Hearing Sciences Research Centre, Anglia Ruskin University, Cambridge, United Kingdom; 3 Department of Psychology, Institute of Psychiatry, Psychology and Neuroscience, King’s College, London, United Kingdom

**Keywords:** para sport, classification, visual impairment, football, paralympic

## Abstract

**Introduction:**

Classification systems aim to minimise the impact of impairment on competition outcome. To measure the effectiveness of a classification system, the relationship between impairment and performance must be investigated. There are two forms of football for athletes with vision impairment (VI): blind football and partially sighted football. Athletes are allocated to either one based on VI severity. Research is yet to assess the impact of impairment on performance in competition; therefore, this study aimed to measure the impairment-performance relationship in male blind, partially sighted and women's blind football.

**Methods:**

Notational data consisting of several technical performance measures were assessed (including, but not limited to, possession, passing, shots, and goals) and combined with visual function data from elite blind and partially sighted footballers. Correlations of notational match data and visual acuity (VA) were measured for male blind and partially sighted footballers (study one) and women's blind footballers (study two).

**Results:**

In study 1: the team-level analysis revealed a weak but statistically significant correlation between win ratio and VA for male blind football (*r* = 0.227). The player-level analysis revealed that VA was correlated with defensive zone clearances (*r* = 0.198), shots on target (*r* = 0.237), and shots saved (*r* = 0.229). In partially sighted football, team-level analysis revealed that VA was significantly correlated with win ratio (*r* = −0.534) and ball possession (*r* = 0.419). The player-level analysis revealed that VA was correlated with the number of fouls committed (*r* = 0.273) and fouls won (*r* = −0.273). These findings suggest that impairment may impact the outcome of competition in male blind and partially sighted football. In study two, win ratio was not correlated with VA (*r* = −0.095) in women's blind football, implying that impairment does not impact competition outcome and that fairness may be achieved.

**Discussion:**

These results evidence a different impairment-performance relationship for each version of the sport, and that the current classification system may not optimise fairness across each form of football.

## Introduction

Classification promotes fair and meaningful competition by minimising the impact of impairment on competition outcome (1). Para Sport classification involves two steps, the first requires individuals to meet the minimum impairment criteria (MIC), the level of impairment at which performance is impacted in the un-adapted form of the sport (1), in the second step athletes are allocated to a class to compete with others of similar activity limitation (2). Footballers with vision impairments (VI) are classified based on their visual acuity (VA; the spatial resolving capacity; higher logMAR values denote poorer vision) and/or visual field (VF; the entire area that can be seen by the eye when fixating at a point; higher values denote better visual function). The current classification system originates from the World Health Organisation's VI and blindness definitions (3) and has historically been implemented within several VI sports. However, footballers can be classified as B1 (VAs >2.6logMAR), B2 (VAs from 1.5 to 2.6logMAR and/or VF <10°) or B3 (VA from 1.0 to 1.4logMAR and/or VF <40°) (4). Class allocation determines whether footballers compete in blind or partially sighted football.

Blind and partially sighted footballers compete in adapted forms of futsal. B1 male footballers compete in blind football and rely on auditory information (verbal instruction from guides, sounded ball, and shouts of “voy” so other players' location can be estimated). Moreover, eyeshades are worn, kickboards are mounted pitch side, and matches are played on an uncovered outdoor court to ensure optimum acoustics (4). Male B2 and B3 footballers compete in partially sighted football, which is played on an indoor court where light of equal intensity is applied to all areas of the court, and teams cannot field more than two B3 players and compete with a sighted goalkeeper (4). Partially sighted footballers often have to adapt to lighting conditions, which can differ considerably between classification and competition venues. Visual pathologies respond differently to lighting conditions; therefore, performance of partially sighted footballers can be significantly impacted by lighting conditions (2). Meanwhile, women footballers compete under blind football rules, irrespective of class allocation but are required to wear eyeshades.

The current classification system is not evidence-based or sport-specific, as it does not account for the impact of impairment on performance in the specific sport (1). Research towards a sport-specific classification system in footballers was commenced with an expert panel consultation including international footballers (current or retired), coaches, classifiers, administrators and referees (5). The expert panel suggested the existing system does not create equitable competition and that those with poorer impairments in the B2 class are unable to compete due to their impairment disproportionately impacting performance, they also believed eyeshades effectively create equitable competition. Moreover, research has measured vision profiles of elite VI footballers (6) and assessed the MIC and vision testing procedures (7, 8). However, research has yet to assess the impact of impairment on performance in competition under the current classification system, which is a necessary step towards sport-specific evidence-based classification systems (9).

Assessing the impairment-performance relationship in competition involves selecting measures of visual function and performance (2). Conflicting methods have been used to assess the impairment-performance relationship in sprinting (10), judo (11), swimming (12), shooting (13), skiing (14) and goalball (15). Studies focusing on individual sports have measured the correlation between impairment and performance (10–14), whereas others measured performance differences among B1, B2 and B3 competitors (15). These discrepancies are due to the complexities of measuring performance in team sports, as no single performance variable is sufficient, due to the multifaceted nature of those sports (15). However, poorer vision has been correlated to poorer swimming, judo and sprinting performance (10–12). A non-significant relationship was observed in shooting, with the best performers typically having worse vision (13). In goalball, where all individuals compete together irrespective of class allocation and wear eyeshades, there were non-significant differences between classes across each performance metric (15).

Tactical, technical and perceptual-cognitive skills were considered critical to winning and are likely impacted by impairment (5). Perceptual-cognitive skills such as orientation, spatial awareness, tracking and movement around the court are not measurable from assessing performance in competition (15). However, work conducted in goalball analysed technical parameters (15). Through the use of notational data, research in traditional futsal has measured an abundance of technical performance indicators, specifically, team metrics such as possession, offensive zone entries, alongside individual metrics, such as passing, shooting, dribbling, ball recoveries (16–18). Findings have often been inconsistent; consequently, there are no universally accepted key performance indicators, nor has research yet identified consistent key performance indicators specifically to each variation of football for those with VI.

In order to assess the impairment-performance relationship in competition, we extracted footballers' VA and VF measurements from the International Blind Sports Federation (IBSA) sport administration system (ISAS) that competed in an IBSA-sanctioned event, similarly to previous classification research (11, 15). This enabled the relationship between notational match data and visual function to be assessed. To identify valid performance metrics, an initial study on matches from the 2023 IBSA world championships was conducted, aiming to identify key performance indicators (19). The findings evidenced different key performance indicators, indicating unique technical and tactical demands within each variation of football (19). Passing efficiency was the greatest predictor of win ratio in male blind and partially sighted football; in both sports, higher passing efficiencies were associated with better outcomes. However, conflicting relationships were observed for possession. Male blind football teams benefited from increased possession as this positively impacted win ratio, indicating a possession-based strategy was optimal. On the other hand, possession negatively impacted the win ratio in partially sighted football, which implied that a counterattacking strategy may be favourable. In contrast, offensive zone (attacking third) entries were the greatest predictor of win ratio in women's blind football; whereas, higher passing efficiencies negatively impacted this metric, suggesting that teams benefited from a direct playing approach. Moreover, when identifying variables that predicted performance (the most important variables to win ratio) in each variation of the sport, again, conflicting performance indicators were identified. In male blind football, successful dribbling had a positive impact on passing efficiency, whereas fouls committed and possessions lost in the defensive half negatively impacted this metric. Whereas, in women's football, possessions lost in the defensive half and unsuccessful set-piece passes negatively impacted offensive zone entries, the number of successful passes positively impacted this metric. In partially sighted football, dribbling and defensive zone clearances predicted passing efficiency and indicated a negative impact. Therefore, due to the diverse key performance indicators across male blind, women's blind and partially sighted football, an exploratory analysis including multiple metrics of performance may be most effective in measuring the impairment-performance relationship in competition.

This exploratory study aimed to assess the impairment-performance relationship of multiple performance metrics and visual function (VA and/or VF) to identify whether performance differed as a function of class allocation within a competitive setting. Previous work has suggested that the current classification system does not create equitable competition (5); therefore, analysis of the impairment-performance relationship could identify whether performance is impacted by impairment and highlight the effectiveness of classification in male blind, women's blind and partially sighted football. To effectively fulfil the aims of the study for each version of the sport, we adopted a multi-study approach; study one included separate analyses of male blind and partially sighted football, whereas study two analysed women's blind football.

## Study 1 method

### Participants

A notational video match analysis of 30 teams was conducted from the 2023 IBSA World Championship. Seventy-two games involving 221 footballers with a VI were played. All matches and teams competing across the male blind and partially sighted football Championship were included in our analysis. Male blind and partially sighted football were analysed separately. *Male blind football:* Fifteen international teams competed in 39 games, including 115 visually impaired footballers. According to recent research there were 417 active elite male blind footballers registered on the ISAS database (6), therefore our sample accounted for approximately 28% of the entire population assessed, and athletes competing were included within this sample. One hundred and five players were included in the analysis; 8 were excluded due to having fewer than five game involvements, and two were excluded because visual function data were unavailable. Of the 105 male B1 footballers (aged 29.47 ± 7.20 years), 46 were B1-light perception (LP), and 59 were B1-no LP (NLP) classified. *Partially Sighted Football:* Seven international teams competed in 15 games, which included 59 visually impaired footballers. Fifty-eight were included in the analysis; 1 player was excluded due to having less than five game involvements. Of the 58 partially sighted footballers (aged 31.22 ± 6.88 years) in our analysis, 23 were classified as B3 and 35 were B2. According to recent research there were 79 active elite male partially sighted footballers registered on the ISAS database (6), therefore our sample accounted for approximately 73% of the entire population assessed, and athletes competing were included within this sample. Fifty-seven partially sighted footballers were classified based on their VA (B2 = 35, B3 = 22), and 1 B3 was classified based on an impaired VF. IBSA granted permission (in writing) for researchers to access the ISAS database for research purposes, similar to previous research (6, 11, 20). Ethical approval was obtained from the lead University ethics committee [ref: ETH2223-8358].

### Squad lists

Squad lists that contained player names and shirt numbers were extracted from the IBSA World Championship tournament website (21).

### Measures of visual function

#### Classification database

The squad lists were cross-referenced with the ISAS database. Classifiers measure an athlete's VA and/or VF. An Early Treatment Diabetic Retinopathy Study Chart (ETDRS) Tumbling E chart is used to measure VA and is recorded in logMAR. For athletes unable to correctly identify any letter on the ETDRS chart, the Berkeley Rudimentary Vision Test (BRVT) is used (4). Goldmann, Humphrey, or Octopus are approved apparatus for measuring VF; however, the software used must assess full fields (80° or more), not only central VF (4).

VI athletes diagnosed with progressive medical conditions causing vision to degrade over time are required to undergo re-evaluation. Depending on their visual condition, classifiers may determine that athletes need to be reclassified within one, two or four years. Alternatively, those diagnosed with non-progressive visual conditions may be given confirmed status and do not require re-evaluation, which typically occurs for athletes with severe vision loss (B1 athletes deemed to have LP or NLP) (11). Therefore, where available classification documents were used to extract visual function data from the 2023 IBSA World Championships, where this was not available, a player's most recent classification assessment was used. This allowed the allocated class (B1, B2 or B3), VA and/or VF to be extracted for each squad member competing. Two male blind footballers were excluded due to their visual function data not being accessible. To allow comparison of footballers classified as B1, they were split into B1-LP and B1-NLP, for the team-level correlation analysis, players who had LP and NLP were assigned a 3.00logMAR and 4.00logMAR, respectively (6, 13, 22).

### Performance variables

Researchers were granted access to the footage of all games played at the 2023 IBSA World Championships by the IBF Foundation (23), which comprised of a pitch-side tactical view. All footage were uploaded to ISportsAnalysis (24), where a notational analysis of each game was conducted. Each game was analysed using a Lenovo laptop (Version 10.026100 Build 26100) at a resolution of 1,920 × 1,080.

This exploratory analysis compared a total of 31 match-related events that an individual could conduct. Match events assessed were selected after reviewing previous investigations on futsal (17, 18, 25) alongside a Delphi study assessing the current classification system (5). Dribbling and passing were analysed as experts believed these attributes were most likely to be impacted by vision loss (5). Other game-related events included were tackling, fouls, shots and attacking half-ball recoveries (indirect and direct). Shots, passing (efficiency and total passes), and dribbling were all significant predictors of match outcome or key performance indicators as identified in an initial study (19). A complete list of match events coded along with the operational definitions can be seen in Table 1. Previous research has divided a player's contribution (number of actions involved in) by the number of minutes played (18). Due to the footage including replays, we were unable to accurately determine the number of minutes played by each player. Therefore, participants were required to have a minimum of 5 game involvements to be included in the analysis.

**Table 1:** Operational definitions that were adapted following extraction from (26).

Variables	Definitions
Possession (%)	Possessions are defined as one or more sequences in a row belonging to the same team. A possession is ended by the opposition gaining control of the ball. A team's match possession was calculated by time in possession of the ball divided by total match time.
Total passes (abs)	The total number of attempts to deliver the ball from one player to another player on the same team. A player can use any part of their body (permitted in the laws of the game) to execute a pass. Goal kicks, corners and free kicks are played as a pass. Crosses, keeper throws, and kick-ins (partially sighted only) were coded as a pass.
Total passes from set pieces (abs)	Total passes that were played through set-pieces (corners, goal kicks, kick-offs, free kicks (direct and indirect) and kick-ins (partially sighted only). A set-piece was defined as any action where the ball starts from a dead-ball situation.
Total open play passes (abs)	The total number of passes attempted that were played in open play (combination of successful and unsuccessful)
Total shots (abs)	The total number of shots taken by a team. This is the combination of shots on target (including goals), shots blocked, shots saved and shots off target.
Attempted dribbles (abs)	This is an attempt by a player to beat an opponent when they have possession of the ball. Attempted dribbles were also coded when the player overran the ball with a heavy touch when trying to take-on an opposition player.
Set piece passing success (%)	Successful set-piece passes divided by the total number of set-piece passes.
Open play passing success (%)	Successful open play passes divided by the total number of open play passes.
Offensive Zone Entries (abs)	When a team/player enters an opponent's defensive zone (which was indicated by the lines on the court) with the ball under control. In blind football, the court is divided into three longitudinal thirds. The offensive zone represents the area where the attacking team's target (goal) is located. In partially sighted football, the court is divided into four longitudinal quarters; the offensive zone is the quarter where the intended target (goal) is located. This is in accordance with official court markings for each version of the sport (4).
Successful pass (abs)	A completed pass is a pass which goes to a teammate directly without a touch from an opposition player.
Unsuccessful pass (abs)	An unsuccessful pass which results in the team losing possession or an opponent touching the ball.
Set piece successful pass (abs)	The number of successful passes that were played through set-pieces (corners, goal kicks, kick-offs, free kicks (direct and indirect) and kick-ins (partially sighted only).
Set piece unsuccessful pass (abs)	The number of unsuccessful passes that were played through set-pieces (corners, goal kicks, kick-offs, free kicks (direct and indirect) and kick-ins (partially sighted only).
Open play successful pass (abs)	The total number of successful passes that were played in open play. This could not be from a set-piece and had to be during active play.
Open play unsuccessful pass (abs)	The total number of unsuccessful passes attempted that were played in open play.
Shots on target (including saved shots) (abs)	A deliberate attempt to score that is on target. Includes all goals being scored and shots on target saved by the goalkeeper. A shot where the goalkeeper prevents the ball from entering the goal with any part of their body when facing an intentional attempt from an opposition player. (1) This includes unintentional or misplaced efforts on target from a goalkeeper's teammates, but only if the intervention is not perceived to be a routine collection of the ball. (2) If, after a Goalkeeper's intervention, a more prominent defensive action from a teammate prevents the ball from entering the goal, this will be categorised as a block for the teammate, not a Save for the goalkeeper. (3) If the ball goes behind the goal because of a goalkeeper intervention, the match officials must award a corner for it to be recognised as a save.
Blocked shot (abs)	A Blocked Shot is defined as an attempt to score, including: (1) An attempt on target blocked by an outfield player, where other defenders or a Goalkeeper are behind the Blocker. (2) Incorporates shots blocked unintentionally by the shooter's teammate. (3) Clearances off the line by an opposition player (last line Blocks) are classified as Shots on Target and not as a Blocked Shot.
Shot off target (abs)	A deliberate attempt to score that misses the target, without contact from a player diverting the ball from on target to off target. A shot hitting the frame of the goal is classified as a shot off target unless the ball subsequently enters the net. A blocked shot is not classified as a shot off target.
Goal (abs)	Attributing a goal to the goal-scoring player, or in the case of an own goal, to the defending player. This occurs when the whole ball crosses the line between the goalposts.
Unsuccessful dribble (abs)	An unsuccessful dribble means the player loses control or possession of the ball when attempting to dribble past (/take on) a player.
Successful Dribble (abs)	A successful dribble means the player beats the defender while retaining possession, or where a player advances with the ball and successfully passes to a teammate or shoots.
Unsuccessful tackle (abs)	An unsuccessful tackle is when a tackle is made but the ball goes to an opposition player, or the opposition player retains possession, or the challenge was classed as a foul.
Successful tackle (abs)	A successful tackle is deemed to be when the tackler or one of their teammates regains possession because of the challenge, or when the ball goes out of play and is safe.
Defensive zone clearances (abs)	Coded when the ball is struck (kicked or headed) in the defensive zone (as indicated by lines on the court) with no clear and obvious intended target.
Attacking half regains (abs)	This is an accumulation of loose ball recovery, ball steal in the attacking half and interceptions in the attacking half.
Loose ball recovery attacking half (abs)	This is where a player recovers a loose ball in the attacking half, where neither team has possession or where the ball has been played directly to him by an opponent, thus securing possession for their team.
Ball steal in attacking half (abs)	Coded when a player regains possession from another player (tackled a player or when the player is taken from a player after a heavy touch). Ball lost in defensive half—when a player is dispossessed in their half (either tackled/unsuccessful pass/lost control of the ball, where the ball has led to a change in team possession)
Intercept in attacking half (abs)	This is where a player intercepts an opponent's pass in the attacking half.For this to be coded, an obvious pass must have been played; if this is not the case, loose ball recovery must be coded.
Possession lost in defensive half (abs)	When a player was tackled in his/her half, it resulted in his team losing possession of the ball. Alternatively, this was coded when a player passed the ball directly to the opposing team.
Foul Committed (abs)	A foul conceded is any infringement penalised as foul play by an official that results in a free-kick or penalty event. Incidents where a match official has played advantage and subsequently cautioned a player do not contribute towards the total foul count for the player or team. In these scenarios, a free-kick or penalty event must occur for a foul to be awarded.
Foul won (abs)	When a player was awarded a free kick or penalty following being fouled.

### Statistical analysis

A chi-square goodness-of-fit test measured whether classes were similarly represented. In blind football, this comparison involved the number of footballers classified as B1-LP vs. B1-NLP, whereas in partially sighted football, it compared the number of B2 and B3 footballers. A significant result (*p* < 0.05) would indicate that the number of players in each category differed significantly.

A Shapiro–Wilk test of normality of each variable examined the distribution of the data. Variables in male blind and partially sighted football were all non-normally distributed, indicated by a *p* < 0.05*.* Mean ± SD were reported for normally distributed variables, whereas medians [interquartile ranges (IQR)] were reported for variables that were not normally distributed.

A team-level analysis consisted of Pearson correlation coefficients (*r*), which measured the relationship between team performance and mean VA. Only VA of players who competed in the game were included in the analysis, providing a mean VA of active players. The performance variables selected were win ratio (the number of wins divided by total games played), goals scored, goals conceded, possession and offensive zone entries. Significant correlations were indicated at a *p* < 0.05*.*

A player-level analysis consisted of Spearman's correlation coefficients (*r)*, which measured the relationship between VA and performance. This allowed the continuous relationship between performance and impairment to be assessed in addition to the differences in performance between classes. A perfect relationship is indicated by a *r* = 1.00, a strong correlation *r* = 0.90–0.70, a moderate relationship *r* = 0.40–0.70, a weak relationship *r* = 0.10–0.30 and no correlation *r* = 0.00 (27). This scale was used for both Spearman and Pearson correlations. Due to few athletes being classified based on an impaired VF, the same analysis could not be conducted.

Male blind football: Mann–Whitney *U* compared the B1-LP and B1-NLP compared the frequency of each variable. The rank-biserial (*r_pb_*) correlation indicated the effect size; a small effect was *r* = 0.10, *r* = 0.30 a medium effect, and *r* = 0.30 a large effect (28).

Partially sighted football: Mann–Whitney *U* compared the B2 and B3 compared the frequency of each variable. The rank-biserial (*r_pb_*) correlation was used as the effect size, where a small effect was *r* *=* 0.10, *r* = 0.30 a medium effect, and *r* = 0.30 a large effect (28). The rank-biserial (*r_pb_*) effect sizes were selected in line with recommendations made in sports science (29); such effect sizes can be simply interpreted and allow for consistency within the sports science literature.

#### Reliability

Intra-class correlations (ICC) were calculated for several variables to evaluate reliability in Study 1 and Study 2. A two-way random effects model was selected to measure the absolute agreement among raters. Ten percent of the matches (4 male blind, 2 women's blind and 2 partially sighted) were retracked by the lead researcher and independently analysed by a second analyst to measure intra- and inter-rater reliability (30). An ICC below 0.5 indicates poor reliability, between 0.5 and 0.75 indicates moderate reliability, 0.75–0.9 indicates good reliability and above 0.9 indicates excellent reliability (31).

Excellent intra-rater was observed for possession [ICC (95%CI); 0.989 (0.968–0.996)], total shots [0.986 (0.962–0.995)], dribbles attempted [0.979 (0.942–0.992)], offensive zone entries [0.978 (0.939–0.993)] and total open play passes [0.992 (0.978–0.997)]. Excellent inter-rater was observed for possession [ICC (95%CI); 0.991 (0.975–0.997)], total shots [0.992 (0.979–0.997)], dribbles attempted [0.941 (0.793–0.981)], and total open play passes [0.967 (0.618–0.992)], whereas a good agreement was observed for offensive zone entries [0.878 (0.102–0.971)].

## Study 1 results

### Chi-Square analysis

The goodness-of-fit analyses did not reveal a significant difference in the number of footballers allocated to each class in blind football [*χ*^2^ (1, *N* = 105) = 1.61, *p* = 0.205, *v* = 0.120] or partially sighted football [*χ*^2^ (1, *N* = 58) = 2.48, *p* = 0.205, *v* = 0.21].

### Team impairment-performance relationship

#### Male blind football

Mean team VA was significantly correlated with win ratio. This relationship indicated that teams with poorer vision (higher logMAR values) had higher win ratios; however, this association was weak. Goals scored, goals conceded, ball possession, and attacking third entries were not significantly correlated with team VA, see Table 2.

**Table 2:** The relationship between mean team visual acuity (VA) and team metrics in male blind and partially sighted football.

Format	Win ratio	Goals scored	Goals conceded	Possession (%)	Offensive zone entries
Male blind football	*r* **=** 0.227*	*r* = 0.015	*r* = 0.092	*r* = 0.198	*r* = 0.081
	*p* **=** **0.046**	*p* = 0.896	*p* = 0.421	*p* = 0.083	*p* = 0.479
Partially sighted football	*r* **=** −0.534*	*r* = 0.075	*r* = 0.038	*r* **=** 0.419*	*r* = 0.345
	*p* **=** **0.002**	*p* = 0.695	*p* = 0.840	*p* **=** **0.021**	*p* = 0.062

Significant Pearson correlations are indicated by bold writing and *with *r*.

#### Partially sighted football

Mean team VA was significantly related to win ratio, which indicated that teams with better vision (lower logMAR values) had a higher win ratio. Mean team VA was significantly related to ball possession, which indicated that teams that fielded players with poorer (higher logMAR values) vision had more possession of the ball. Both win ratio and possession were moderately related to mean VA (see Table 2). Goals scored, goals conceded and attacking third entries were not significantly related to mean team VA (see Table 2).

### Player impairment-performance relationship

#### Male blind football

VA was significantly related to the number of shots on target, shots saved, and defensive zone clearances, all of which were weak correlations (see Table 3).

**Table 3:** The relationship between visual acuity (VA) and each variable assessed in male blind football.

Variable	VA	
	*r*	*p*
Total open play passes	0.078	0.428
Success open play passes	0.070	0.479
Unsuccessful open play passes	0.106	0.284
Open play passing success (%)	−0.060	0.540
Total shots	0.173	0.078
Blocked shots	0.051	0.606
Shots on target	***0*** **.** ***237***	*0*.015*
Goals	0.185	0.059
Shot off target	0.147	0.135
Saved shots	***0*** **.** ***229***	***0*** **.** ***019*** *
Attempted dribbles (take-ons)	0.087	0.377
Dribbling successful	0.083	0.399
Dribbling unsuccessful	0.040	0.685
Tackles lost	0.095	0.333
Tackles won	0.022	0.826
Defensive zone clearances	***0*** **.** ***198***	***0*** **.** ***043*** *
Fouls committed	0.068	0.493
Fouls won	0.090	0.362
Attacking half regains	0.111	0.259
Interceptions (Att. Half)	0.180	0.066
Loose ball recovery (Att. half)	0.086	0.380
Ball steals (Att. half)	0.095	0.334
Possession lost (Def. half)	−0.105	0.287
Goal efficiency	0.171	0.082

Significant Spearman correlations are indicated by bold writing and *with *p*-value.

**Figure 1:**
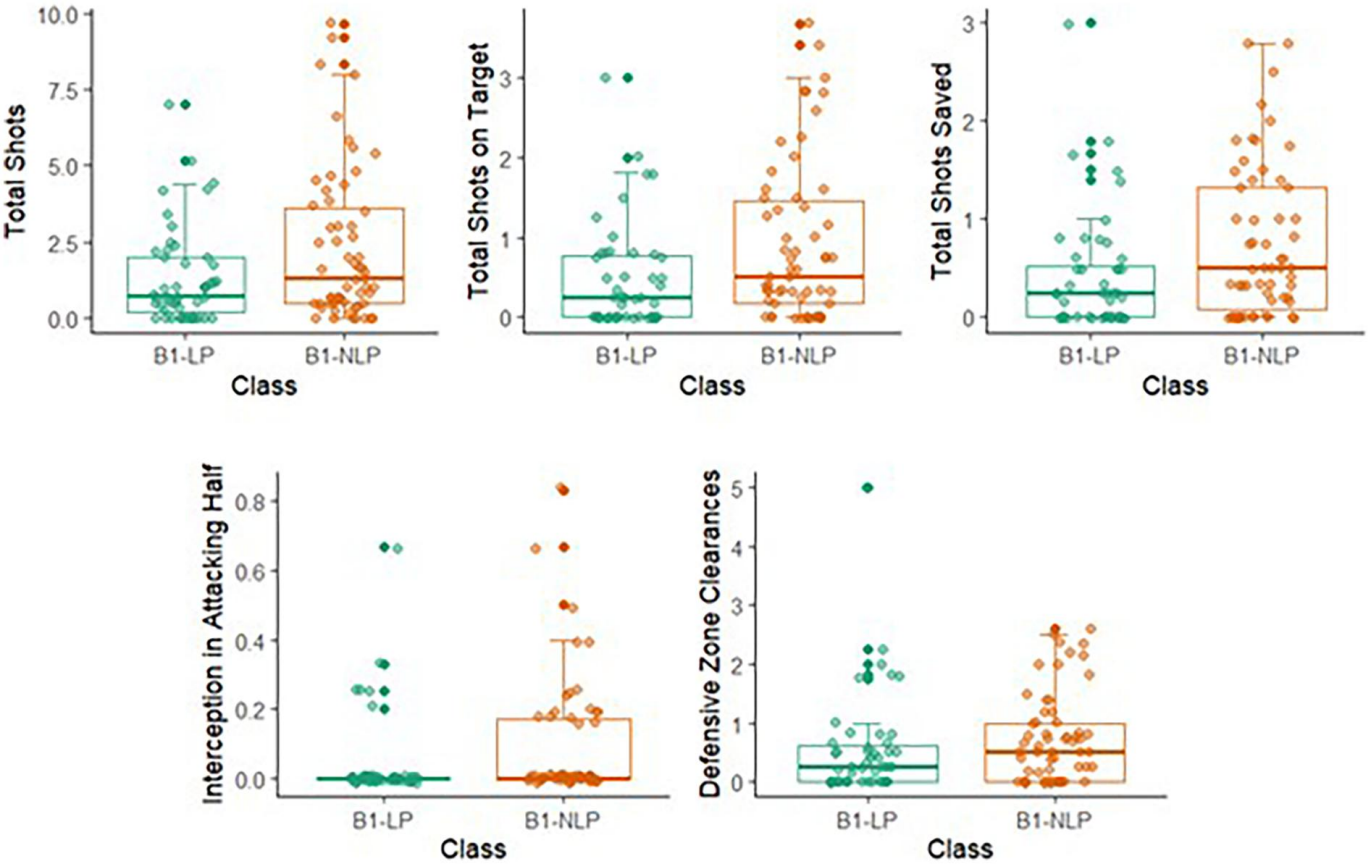
The number of total shots, shots on target, saved shots, interceptions in the attacking half and clearances from the defensive zone for players classified as B1-LP and B1-NLP in male blind football.

B1-NLP footballers took significantly more total shots, blocked shots, shots saved, attacking half interceptions and defensive zone clearances compared to B1-LP footballers. All other comparisons were non-significant. Refer to Table 4 for the complete data and statistics. See Figure 1 for visualisation of variables that significantly differed.

**Table 4:** Median and IQR of each variable compared for B1-LP and B1-NLP footballers in male blind football.

Variable	B1-LP [Median (IQR)]	B1-NLP [Median (IQR)]	*U*	*p*	*r_pb_* effect size
Passing					
Total passes	3.71 [6.58]	5.75 [5.80]	1,217.00	0.368	−0.103
Successful passes	2.00 [4.30]	3.20 [3.50]	1,232.00	0.421	−0.090
Unsuccessful passes	1.42 [1.65]	2.00 [2.24]	1,175.00	0.241	−0.134
Passing efficiency (%)	62.02 [18.64]	60.00 [19.74]	1,468.00	0.575	0.082
Shooting					
Total shots	0.71 [1.68]	1.33 [3.09]	1,045.00	**0**.043*	−0.230
Shots on target	0.25 [0.75]	0.50 [1.28]	973.50	**0**.012*	−0.283
Shots saved	0.25 [0.50]	0.50 [1.25]	985.50	**0**.015*	−0.274
Blocked shots	0.23 [0.50]	0.25 [0.71]	1,248.00	0.474	−0.080
Shots off target	0.29 [0.60]	0.50 [1.21]	1,080.50	0.071	−0.204
Goals	0.00 [0.00]	0.00 [0.25]	1,118.50	0.056	−0.176
Goal efficiency (%)	0.00 [0.00]	0.00 [5.56]	1,137.00	0.079	−0.162
Dribbling					
Total dribbles	2.84 [4.89]	3.00 [5.73]	1,200.00	0.312	−0.116
Unsuccessful dribbles	1.67 [3.39]	2.00 [2.83]	1,269.50	0.574	−0.064
Successful dribbles	1.00 [2.20]	1.33 [2.92]	1,215.00	0.359	−0.105
Tackling					
Tackles won	2.00 [3.04]	2.40 [2.70]	1,298.50	0.708	−0.043
Tackles lost	0.19 [0.48]	0.25 [0.50]	1,209.50	0.326	−0.109
Fouls committed	0.55 [0.75]	0.50 [1.04]	1,250.00	0.490	−0.079
Fouls won	0.50 [1.00]	0.67 [0.78]	1,191.50	0.282	−0.122
Ball regains					
Interceptions (Att. Half)	0.00 [0.00]	0.00 [0.17]	1,130.00	**0**.040*	−0.167
Defensive zone clearances	0.25 [0.58]	0.50 [1.00]	1,034.50	**0**.034*	−0.238
Loose ball recoveries (Att. half)	0.25 [1.00]	0.60 [0.82]	1,175.50	0.238	−0.134
Ball steals (Att. half)	0.25 [0.77]	0.33 [0.73]	1,195.00	0.289	−0.119
Attacking half regains	0.59 [1.62]	1.00 [1.50]	1,139.50	0.160	−0.160
Possession lost (Def. half)	0.82 [0.73]	0.60 [0.91]	1,492.50	0.381	0.100

Non-normal distributions were observed for all variables; therefore, non-parametric testing was used to assess differences between classes. Median [IQR], therefore, *U*, *p* and *r_pb_* values were reported.

The table also includes the *U*, *p* and *r_pb_* value.

Significant differences are indicated by bold writing and *with *p*-value.

#### Partially sighted football

VA was significantly correlated with the number of fouls committed and fouls won. There were non-significant relationships with other variables assessed, see Table 5.

**Table 5:** The relationship between visual acuity (VA) and each variable assessed in partially sighted football.

Variable	VA	
	*r*	*p*
Total open play passes	−0.101	0.450
Success open play passes	−0.078	0.562
Unsuccessful open play passes	−0.186	0.163
Open play passing success (%)	0.122	0.361
Total shots	−0.196	0.139
Blocked shots	−0.235	0.076
Shots on target	−0.115	0.391
Goals	−0.120	0.370
Shot off target	−0.136	0.310
Saved shots	−0.123	0.357
Attempted dribbles (take-ons)	−0.156	0.242
Dribbling successful	−0.203	0.127
Dribbling unsuccessful	−0.079	0.553
Tackles lost	−.170	0.201
Tackles won	−0.072	0.590
Defensive zone clearances	−0.242	0.067
Fouls committed	***0*** **.** ***273***	***0*** **.** ***038*** *
Foul won	**−*0*** **.** ***273***	***0*** **.** ***038*** *
Attacking half regains	−0.127	0.341
Interceptions (Att. Half)	0.085	0.525
Loose ball recovery (Att. half)	−0.163	0.222
Ball steals (Att. half)	−0.157	0.241
Possession lost (Def. half)	0.085	0.528
Goal efficiency	−0.006	0.962

Significant Spearman correlations are indicated by bold writing and *with *p*-value.

**Figure 2:**
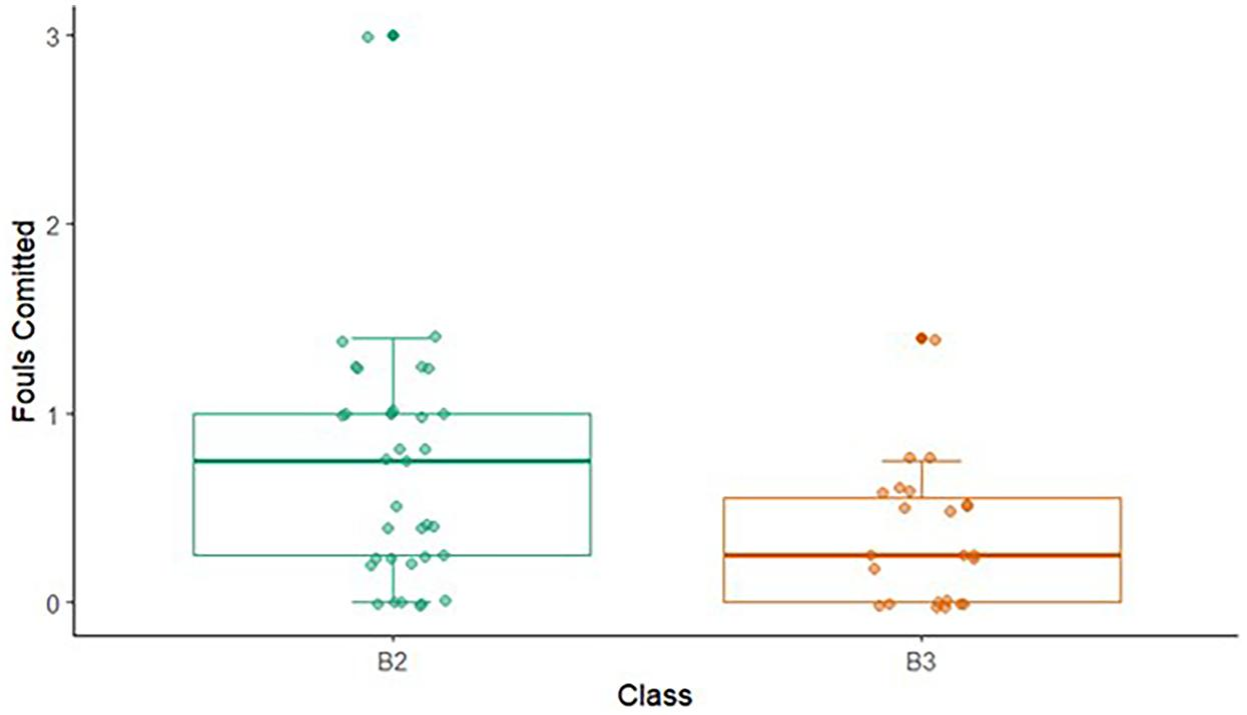
The number of fouls committed by footballers classified as B2 and B3 in partially sighted football.

The number of fouls committed was significantly greater within the B2 class, compared with B3 footballers. All other comparisons were non-significant. Refer to Table 6 for the complete data and statistics. See Figure 2 for visualisation of variables that significantly differed.

**Table 6:** Median and IQR of each variable compared for B2 and B3 partially sighted footballers.

Variable	B2 [Median (IQR)]	B3 [Median (IQR)]	*U*	*p*	*r_pb_* effect size
Passing					
Total passes	26.75 [34.00]	33.60 [22.26]	387.50	0.818	−0.037
Successful passes	20.00 [28.63]	27.60 [19.92]	388.50	0.830	−0.035
Unsuccessful passes	6.50 [5.10]	6.75 [4.55]	372.00	0.633	−0.076
Passing efficiency (%)	79.17 [13.91]	76.43 [12.79]	440.00	0.556	0.093
Shooting					
Total shots	3.75 [4.58]	4.25 [7.58]	375.00	0.668	−0.068
Blocked shots	1.00 [1.68]	1.50 [2.17]	345.50	0.368	−0.142
Shots on target	1.60 [1.95]	1.33 [3.38]	413.00	0.874	0.026
Shots off target	1.00 [1.63]	1.00 [1.88]	387.50	0.817	−0.037
Shots saved	1.40 [1.98]	1.25 [2.28]	399.50	0.968	−0.007
Goals	0.20 [0.45]	0.00 [0.63]	399.00	0.960	−0.009
Goal efficiency (%)	3.33 [6.74]	0.00 [6.36]	445.00	0.484	−0.106
Dribbling					
Attempted dribbles	1.40 [2.21]	1.25 [2.38]	373.50	0.650	−.072
Unsuccessful dribbles	1.00 [1.38]	0.75 [1.21]	410.50	0.905	0.020
Successful dribbles	0.40 [0.68]	0.50 [1.40]	349.50	0.399	−0.132
Tackling					
Successful tackles	2.00 [2.03]	2.20 [1.43]	384.50	0.781	−0.045
Unsuccessful tackles	0.00 [0.25]	0.20 [0.33]	314.00	0.133	−0.220
Fouls committed	**0.75 [0.75]**	**0.25 [0.55]**	**548** **.** **00**	0.020*	**0** **.** **361**
Fouls won	0.25 [0.30]	0.50 [0.75]	304.50	0.117	−0.243
Ball regains					
Interceptions (Att. Half)	0.00 [0.20]	0.00 [0.23]	423.00	0.711	−0.051
Loose ball recoveries (Att. half)	0.20 [0.29]	0.25 [0.58]	301.00	0.095	−0.252
Ball steals (Att. half)	0.20 [0.60]	0.25 [0.45]	378.50	0.699	−0.060
Attacking half regains	0.60 [0.80]	0.67 [1.16]	1,165.00	0.215	−0.141
Possession lost (Def. half)	0.67 [0.80]	0.25 [0.50]	510.00	0.085	0.267
Defensive zone clearances	0.40 [0.80]	0.40 [0.75]	364.50	0.543	−0.096

Non-normal distributions were observed for all variables; non-parametric testing assessed differences between classes. Median [IQR], *U*, *p* and *r_pb_* values were reported.

The table also includes the *U*, *p* and *r_pb_* value. Significant differences are indicated by bold writing and * with *p*-value.

## Study 1 discussion

Study 1 aimed to measure the relationship between impairment and performance through combining technical performance variables and visual function data from the male blind and partially sighted 2023 World Championship. Our findings indicate that the level of impairment and performance were related when assessing at the team-level and the player-level. Male blind teams with poorer VA had higher win ratios; this trend was also observed when assessing individual performance, as those classified as B1-NLP performed more shots, interceptions, and clearances. Alternatively, partially sighted teams with poorer vision corresponded to lower win ratios but higher rates of possession. The player-level analysis highlighted that B2 players committed more fouls compared to B3 footballers.

The findings highlight contrasting relationships between VA and performance; however, the analysis of male blind and partially sighted football suggests that impairment and performance are related and that specific impairment severities may gain a competitive advantage under the existing classification system. Male blind football contradicts much of the literature observing the impairment-performance relationship across multiple sports, which may be attributed to those with poorer visual function having a greater ability to utilise auditory information (32, 33), this being pivotal due to the use of eyeshades in competition. In contrast, the relationship observed in the partially sighted was consistent with much of the literature, where poorer performance was associated with poorer vision (10, 11, 14). This highlights the unique impairment-performance relationships across football and implies that equitable competition may not be attained in male formats of football.

## Study 2 method

The methods used in study 2 were identical to those used in study 1, unless otherwise stated in the methods section below.

### Participants

Eight international women's teams competed in 18 games of the 2023 IBSA World Championship. All teams competing and matches played during the women's blind football World Championship were included in our analysis. The sample included 47 visually impaired footballers. According to recent research, there were 66 active elite female blind footballers registered on the ISAS database (6), therefore our sample accounted for approximately 71% of the entire population assessed, and all athletes competing were included within this sample. Forty-one were included in our analysis, and six were excluded due to having fewer than five game involvements. Of 41 female blind footballers (aged 25.88 ± 7.60 years), 11 were B3, 10 were B2, 11 were B1-LP, and 9 were classified as B1-NLP. Forty female footballers were classified based on an impaired VA (B1-NLP = 9, B1-LP = 11, B2 = 10, B3 = 10), and 1 B3 athlete was classified based on an impaired VF.

### Statistical analysis

A chi-square goodness-of-fit test measured whether classes were similarly represented. In women's blind football, this comparison involved the number of footballers classified as B1-LP, B1-NLP, B2 and B3 footballers. A significant result (*p* < 0.05) would indicate that the number of players in each category differed significantly.

A Shapiro–Wilk test of normality of each game event examined the distribution of the data within each version of the sport. Women's blind football analysis revealed that open play passing success (%) and possessions lost in defensive half were normally distributed (*p* > 0.05); however, all other variables were not normally distributed (*p* < 0.05). Mean ± SD descriptives were reported for normally distributed variables, whereas medians [interquartile ranges (IQR)] were reported for variables that were not normally distributed.

For variables that violated the assumption of normality, main effects were identified by Kruskal–Wallis tests that compared the mean frequency of each variable selected. Effect sizes were calculated using eta squared (small effect *η*^2^ = 0.01, medium *η*^2^ = 0.06, and large *η*^2^ = 0.14) (34).

Normally distributed variables [open play passing success (%) and possession lost in defensive half] were analysed using a one-way ANOVA, where effect sizes were calculated using partial eta squared $\left(\eta_{p}^{2}\right)$ and was interpreted similarly to eta squared (34). Bonferroni follow-up analysis was used to control for multiple comparisons and identify group differences between B3, B2, B1-LP and B1-NLP footballers. The adjusted Bonferroni corrected significance level was accepted at *p* ≤ 0.013, which was planned for parametric and non-parametric testing. *Post hoc* effect sizes calculated using Cohen's *d* (*d*).

The partial eta squared $\left(\eta_{p}^{2}\right)$, eta squared *η*^2^ and Cohen's *d* effect sizes were reported in accordance with recommendations for the sport science literature (29); such effect sizes can be simply interpreted and allow for consistency within the sports science literature.

#### Reliability

Refer to the reliability measures outlined in the methods section of Study 1.

## Study 2 results

### Chi-square analysis

The goodness-of-fit analyses did not reveal a significant difference in the number of footballers allocated to each class in women's blind football [*χ*^2^ (2, *N* = 41) = 0.27, *p* = 0.966, *v* = 0.11].

### Team impairment-performance relationship

Mean team VA was not significantly correlated with win ratio, ball possession, goals scored, goals conceded or offensive zone entries, see Table 7.

**Table 7:** The relationship between mean team visual acuity (VA) and team metrics in womens blind football.

Format	Win ratio	Goals scored	Goals conceded	Possession (%)	Offensive zone entries
Women's blind football	*r* = −0.095	*r* = −0.053	*r* = 0.174	*r* = 0.055	*r* = 0.034
	*p* = 0.581	*p* = 0.757	*p* = 0.311	*p* = 0.752	*p* = 0.845

### Player impairment-performance relationship

Non-significant relationships were identified between VA and the variables measured in women's blind football. The complete list of the correlations can be seen in Table 8.

**Table 8:** The relationship between visual acuity (VA) and each variable assessed in women's blind football.

Variable	VA	
	*r*	*p*
Total open play passes (abs)	0.226	0.155
Success open play passes (abs)	0.245	0.122
Unsuccessful open play passes (abs)	0.158	0.324
Open play passing success (%)	0.184	0.251
Total shots	0.114	0.477
Blocked shots	0.092	0.568
Shots on target	0.115	0.473
Goals	−0.032	0.843
Shot off target	0.054	0.737
Saved shots	0.118	0.461
Attempted dribbles (take-ons)	0.117	0.465
Dribbling successful	0.050	0.757
Dribbling unsuccessful	0.181	0.258
Tackles lost	−0.161	0.316
Tackles won	0.116	0.471
Defensive zone clearances	0.119	0.457
Fouls committed	0.010	0.949
Fouls won	0.141	0.379
Attacking half regains	0.109	0.498
Interceptions (Att. Half)	0.197	0.218
Loose ball recovery (Att. half)	0.127	0.430
Ball steals (Att. half)	0.062	0.698
Possession lost (Def. half)	0.072	0.654
Goal efficiency	−0.050	0.754

There were no significant differences when comparing each class. See Table 9 for full data and statistics.

**Table 9:** Median and IQR of variables that were not normally distributed were compared for women's blind footballers classified as B1-NLP, B1-LP, B2 and B3, for these variables *H*, *p*, and *h* $\eta^{2}$ value. Mean ± standard deviation reported for normally distributed variables, where *F*, *p*, and were reported.

Variable	B3	B2	B1-LP	B1-NLP	Test statistic	*p*	Effect size
Passing							
Total passes	3.40 [5.85]	2.125 [3.94]	3.75 [3.55]	6.00 [4.75]	H(3) = 7.301	0.063	0.113
Successful passes	2.25 [3.85]	1.050 [1.66]	1.75 [1.28]	2.50 [2.33]	H(3) = 6.556	0.087	0.094
Unsuccessful passes	2.20 [2.00]	1.00 [1.95]	2.00 [2.25]	2.75 [3.33]	H(3) = 4.863	0.182	0.049
Passing efficiency (%)	51.79 ± 16.33	34.38 ± 26.65	57.29 ± 17.63	53.57 ± 18.45	F(3,37) = 2.618	0.065	0.175
Shooting							
Total shots	2.00 [5.00]	0.13 [0.44]	1.60 [2.65]	0.40 [8.05]	H(3) = 4.901	0.179	0.050
Shots on target	0.50 [2.13]	0.00 [0.00]	0.50 [1.03]	0.20 [1.55]	H(3) = 6.873	0.076	0.102
Blocked shots	0.25 [0.45]	0.00 [0.15]	0.20 [0.15]	0.00 [1.80]	H(3) = 4.105	0.250	0.029
Shots off target	1.00 [2.20]	0.10 [0.25]	0.75 [1.38]	0.20 [3.80]	H(3) = 3.720	0.293	0.140
Saved shots	0.50 [1.53]	0.00 [0.00]	0.50 [0.93]	0.20 [1.55]	H(3) = 6.687	0.083	0.097
Goals	0.00 [0.20]	0.00 [0.00]	0.00 [0.00]	0.00 [0.00]	H(3) = 5.446	0.142	0.064
Goal efficiency (%)	0.00 [4.03]	0.00 [0.00]	0.00 [0.00]	0.00 [0.00]	H(3) = 5.243	0.155	0.059
Dribbling							
Attempted dribbles	3.60 [5.03]	1.21 [2.26]	4.00 [4.70]	3.00 [9.40]	H(3) = 5.533	0.137	0.067
Successful dribbles	1.50 [2.78]	0.54 [0.94]	1.33 [2.23]	1.00 [7.40]	H(3) = 5.104	0.164	0.055
Unsuccessful dribbles	1.75 [2.00]	0.80 [1.58]	2.75 [3.99]	1.80 [2.00]	H(3) = 4.370	0.224	0.036
Tackling							
Successful tackles	3.25 [2.95]	1.75 [1.46]	2.75 [1.33]	2.50 [3.20]	H(3) = 4.567	0.206	0.041
Unsuccessful tackles	0.20 [0.42]	0.10 [0.36]	0.00 [0.23]	0.00 [0.20]	H(3) = 1.718	0.633	−0.034
Fouls committed	0.25 [0.80]	0.25 [0.38]	0.25 [0.90]	0.60 [1.00]	H(3) = 0.168	0.983	−0.075
Fouls won	0.40 [0.75]	0.00 [0.25]	0.50 [0.51]	0.33 [0.40]	H(3) = 4.568	0.206	0.041
Ball regains							
Attacking half regains	2.00 [4.20]	0.59 [0.69]	2.50 [3.22]	1.33 [3.30]	H(3) = 5.496	0.139	0.066
Interceptions (Att. Half)	0.00 [0.23]	0.00 [0.00]	0.00 [0.42]	0.00 [0.33]	H(3) = 1.654	0.647	−0.035
Loose ball recoveries (Att. half)	1.00 [2.10]	0.33 [0.43]	1.60 [2.58]	0.50 [2.27]	H(3) = 5.637	0.131	0.069
Ball steals (Att. half)	0.75 [2.00]	0.13 [0.44]	1.00 [1.33]	0.67 [1.20]	H(3) = 4.840	0.184	0.048
Possession lost (Def. half)	1.72 ± 0.92	1.51 ± 1.02	1.77 ± 1.07	1.88 ± 1.43	F(3,37) = 0.193	0.901	0.175
Defensive zone clearances	0.60 [1.10]	0.13 [0.55]	0.40 [0.90]	0.40 [1.60]	H(3) = 1.654	0.647	−0.035

Open play passing success and possessions lost (Def. half) were normally distributed variables, where parametric tests were used to assess their significance. The mean ± standard deviation is reported for normally distributed variables, alongside *F*, *p*, and $\eta_{p}^{2}$. All other variables were non-normally distributed, and non-parametric tests were used and medians [IQR] *H*, *p*, and $\eta^{2}$ values were reported.

## Study 2 discussion

Study 2 aimed to measure the relationship between impairment and performance during competition through combining technical performance variables and visual function data from the women's 2023 World Championship. Our findings evidenced that each class performs similarly and that impairment is not related to performance in team or player-level metrics. These findings may be considered somewhat surprising, as previous research has raised possible inequalities within women's blind football due to all classes competing together, for instance, B1 players were considered to have better orientation, spatial awareness and tracking, whereas B2 and B3 footballers were assumed to have better technical attributes such as dribbling, passing, shooting and running (35). However, the findings correspond with goalball, where performance did not differ between player class allocation (15). The adaptations of the sport are similar as players compete together irrespective of class allocation and wear eyeshades; the results indicate that the use of eyeshades effectively creates equitable competition, as suggested by an expert panel (5).

## General discussion

The purpose of this study was to measure the impact of impairment on football performance during competition with adaptations in male blind, women's blind and partially sighted football. These distinct versions of football are considered different sports (4) and were analysed separately. Within male blind football, poorer vision corresponded with higher win ratios. Furthermore, B1-NLP footballers performed more shots, interceptions, and clearances. Within partially sighted football, poorer vision corresponded to lower win ratios, and B2 players committed more fouls compared to B3 footballers. These findings may indicate that some impairment severities gain a competitive advantage under the existing classification system in football. In contrast, non-significant relationships and differences between classes across all performance metrics were observed in women's blind football, which may indicate that eyeshades effectively contribute to equitable competition. Moreover, different impairment-performance relationships were observed in each version of the sports, highlighting the unique impact of impairment on performance.

Our findings highlight that B1-NLP male blind footballers performed more shots, interceptions and clearances, which contributed to higher win ratios and may be an indicator that those players are more comfortable when wearing eyeshades. Although these results are similar to those observed in shooting (13), this relationship may be surprising, as multiple studies observed a negative impact of severe VI on performance (10, 11, 14). It has been suggested that individuals with poorer vision exhibit better cognitive-perceptual skills when wearing eyeshades in football, whereas those with better vision are associated with higher technical skills (35). This raises a potential bias in our analysis, as measuring the frequency of technical performance metrics may favour those with poorer vision, as the frequency of contributions is likely inherently impacted by perceptual cognitive skills. Therefore, those with poorer vision who have a greater sensory adaptation and improved perceptual-cognitive skills are likely to contribute more often.

The impact of congenital vs. acquired VI must be considered. Those with a congenital VI are believed to develop superior auditory and tactile senses (36), leading to better perceptual-cognitive skills (orientation, tracking and movement around the court). In contrast, those with acquired VIs are reliant on their “visual memory”, which is advantageous for technical actions due to visual modelling (35, 36). Despite this, if a footballer has poor perceptual-cognitive skills, their technical ability is unlikely to significantly influence a game, due to an inability to locate the ball and affect the game. This highlights the importance of sensory adaptation, as football performance is profoundly impacted by orientation, tracking, and movement around the court. Future research may investigate the effects of acquired or congenital factors, including age at which they started the sport, training experience, years competing, and total training hours, similar to previous work (14). Whether a VI is acquired or congenital is not recorded during classification; therefore, we were unable to include it in our analysis. Sensory adaptation is imperative in football, due to the availability of an abundance of auditory information.

Alternatively, the quality of auditory information is likely to have a significant impact; therefore, a guide's ability to communicate effectively will have a profound effect on the performance of blind footballers, particularly in executing offensive and defensive strategies. Auditory information (ball, guides, and shouts of “voy”) when blind football teams are not in possession may be considered of heightened importance, as misinterpretations may compromise defensive organisation. However, we are unable to find research assessing the impact of guides on football performance or tactical execution.

Research has shown that blind footballers are more precise and decisive in identifying auditory signals compared to sighted and blind non-athletes (33). Furthermore, blind footballers were correct more often and assertive in their responses when locating a sound's origin (32). Although LP and NLP were not directly compared, it does suggest that those with poorer vision may utilise auditory information more effectively and implying more profound sensory adaptive response (37). This improvement is attributed to the neuroplasticity (adaptation of neuronal networks in the brain in response to changing environmental conditions), which allows multisensory integration (where the brain integrates information from multiple sensory modalities to enhance perception) (38). Individuals diagnosed with VI are subject to various adaptations (physiological and neurological), such adaptations are greater in magnitude for those who experienced early blindness, leading to a higher degree of neuroplasticity allowing greater integration of non-visual senses (37). Those who experienced early blindness and/or have poorer vision (i.e., B1-NLP) may be advantaged in football due to more established multisensory function which occurs due to adaptations within the brain (37 for an in-depth review), possibly explaining why those with poorer vision performed more shots, interceptions, clearances, and achieved higher team win ratios. Effective use of auditory information can be improved through training (33); therefore, confounding variables will likely impact this.

The impairment-performance relationship in partially sighted football supports the suggestion that the classification system does not create equitable competition (5). Better vision was associated with higher win ratios, implying that impairment does impact competition outcome. A similar relationship has been observed in VI sprinting and judo (10, 11). Additionally, teams with better vision had lower levels of ball possession. The importance of ball possession in futsal has been highly debated, with some studies suggesting it is a key performance indicator (39), whereas other research suggests that its importance is limited (17, 18, 40). It is plausible that holding possession of the ball may negatively impact counterattacks, which are key strategies in partially sighted football (19). Counterattacks aim to exploit unstructured opposing defences caused by the absence of the adversary's collective fallback, where speed and depth predominate in the game after ball recovery (41, 42) and are associated with enhanced ball possession effectiveness and outcomes (18, 42). Therefore, controlling possession of the ball may not be optimal in partially sighted football and may decrease offensive efficiency.

The correlation analysis revealed that those with poorer vision committed more fouls and were fouled less often; this corresponded to B2 footballers committing significantly more fouls. This finding aligns with previous Para sport research, which has shown that performance declines as the impairment becomes more severe (10, 11, 14). This may indicate a difficulty tracking multiple objects (ball and player) while in motion, which corresponds to previous research that highlighted an impaired VA reduces performance on a multiple-object tracking task, which may explain why B2 players committed more fouls (43). Despite this, we are unable to determine whether this finding can be attributed to B2 footballers fulfilling more defensive roles, where tackling is attempted more frequently. As previous work has classed set pieces as effective and efficient scoring opportunities (41), the ability to win fouls may be critical, and coaches must look to optimise set pieces as they enable coordinated attacks, where preplanned actions and movements can be implemented (18). These findings indicate that the impairment-performance relationship of motion perception and dynamic VA warrants further investigation (5) and that the impact of impairment on defensive performance needs to be measured, as work to date has focused on anticipation (computer assessment) and while in possession of the ball (8).

Analysis of women's blind football suggested that impairment and performance were not related, which indicates that measures such as eyeshades contribute to equitable competition. This relationship contradicts other VI sports that do not use eyeshades and have reported significant correlations (10, 11, 14). Moreover, our results oppose study one's findings, where significant differences and relationships were observed, which indicates that the use of eyeshades and allowing all classes to compete together impacts the impairment-performance relationship during competition. The non-significant relationship and differences are comparable to those published assessing the impairment-performance relationship in goalball (15). These findings suggest that eyeshades are an effective method to create equitable competition in VI sport, as suggested by an expert panel (5). Should women's blind footballers continue to compete together irrespective of class allocation, the critical aspect of classification is the MIC to ensure only those whose impairments impact performance are eligible (7, 8).

The study analysed footage of the IBSA World Championship that was streamed for public viewing; therefore, we were unable to calculate the number of minutes played per player, resulting in the comparison of unstandardised performance metrics, failing to account for the impact of playing time. The player's position was not accounted for, as we were unable to identify it conclusively, because tactical information (i.e., the intended player position) developed by coaches was not available. However, the link between VI and position is an interesting consideration; visual profiles may influence a player's position. For instance, different visual constraints impact gaze behaviour differently in VI athletes (44). Therefore, certain visual profiles may favour the demands of a specific position. For instance, defenders often anchor their gaze on a particular point while monitoring their environment through peripheral vision (45); therefore, an impaired VF may cause defensive situations to be more challenging and profoundly impact performance. Researchers may record gaze behaviours across multiple situations in VI footballers, which was beyond the scope of the present study.

Our analysis consisted of one tournament; therefore, future work should monitor the impact of impairment on performance in future competitions to understand whether our findings are representative of the general trend in blind and partially sighted football. Therefore, this may be considered a significant limitation as the findings provide a snapshot of the impairment-performance relationship in football. Comparable research assessing VI Judo (46) and goalball (15) has analysed multiple competitions; it may be necessary to combine the data from the present study with future competitions to monitor the trends observed over a longer period. The sample size observed in women's blind and partially sighted compared to male blind football was much smaller (number of games and players), which may have impacted the sensitivity of our analysis, causing a small effect to be unidentified; therefore, this may not be a full representation of the impairment-performance relationship within these formats. Confounding variables such as acquired vs. congenital impairment, age started the sport, training experience, years competing and total training hours (14, 47), which likely impact the impairment-performance relationship, could not be accounted for. Moreover, key skills such as spatial awareness, anticipation, decision making and movement were not measurable. Previous work has highlighted the importance of such attributes (35, 48) and suggested that impairment would likely affect such skills (5). Therefore, the impact of performance on those skills could not be accounted for within our analysis.

To summarise, male blind teams with poorer vision had higher win ratios. The player-level analysis supports this as B1-NLP footballers actioned more shots, interceptions and clearances. These findings suggest that those who cannot perceive light may have an advantage, which could be due to better orientation, spatial awareness and tracking (35), as well as being more effective in their use of auditory information. The impairment-performance relationship observed in male blind football is similar to that of VI shooting, where athletes largely rely on auditory information (13). It should be noted that this relationship differs from VI judo, skiing, and sprinting (10, 11, 14) events, which are determined by an individual's technical performance. Football may be considered a more unpredictable environment compared with individual sports, and blind footballers' game involvement is likely reliant on the ability to process auditory information. Contrastingly, the adaptations and classification structure in women's blind football led to a non-significant relationship between win ratio and impairment and non-significant differences between classes. This suggests that the adaptations appear to create equitable competition, and the impact of impairment is minimised. Alternatively, teams in partially sighted football who fielded players with poorer vision had lower win ratios. In contrast, player-level analysis revealed that players with poorer vision committed more fouls and won fewer fouls. These findings suggest a performance advantage for those with better vision. In each variation of the sport, attributes such as dribbling and passing were not impacted by impairment; these were skills that were suggested to be critical to winning and likely to be impacted by impairment, according to an expert panel (5). Despite this, on a team level, the impairment is associated with win ratio, indicating that equitable competition may not be achieved in male blind and partially sighted football. Our evidence suggests that equitable competition was achieved in women's blind football due to non-significant relationships and differences.

## Data Availability

To ensure confidentiality of visual function data for those included within our analysis, we are unable to share the data. Further inquiries can be directed to the corresponding author.
